# The Short-Term Kinetics of sICAM-1 after Induction of Acute Experimental Pain in Healthy Volunteers

**DOI:** 10.3390/jcm10092021

**Published:** 2021-05-09

**Authors:** Philipp Lüke, Eduard Kraft, Shahnaz Christina Azad

**Affiliations:** 1 Department of Anaesthesiology, University Hospital, Ludwig-Maximilians-University Munich, 81377 Munich, Germany; Shahnaz.Azad@med.uni-muenchen.de; 2 Interdisciplinary Pain Center, University Hospital, Ludwig-Maximilians-University Munich, 81377 Munich, Germany; 3 Department of Orthopedics, Physical Medicine and Rehabilitation, University Hospital, Ludwig-Maximilians-University Munich, 81377 Munich, Germany; Eduard.Kraft@med.uni-muenchen.de

**Keywords:** sICAM-1, QST, acute pain model, biomarker, heat pain, cold pain

## Abstract

Intercellular adhesion molecule-1 (ICAM-1) mediates extravasation of leukocytes, releasing proinflammatory cytokines or endogenous opioids in the inflamed tissue. Thus, ICAM-1 is a crucial component of peripheral antinociception. Previously, we demonstrated a significant correlation between the soluble form of ICAM (sICAM-1) in serum and pain intensity reported by chronic pain patients. The present study examines the role and kinetics of sICAM-1 in experimentally induced acute pain. Three groups of 10 subjects were exposed to 10 min of high (capsaicin-enhanced) or low-intensity heat pain or cold pain, respectively. Thermal stimuli were induced using a device for quantitative sensory testing. Topical capsaicin significantly increased heat pain intensity without the risk of thermal tissue damage. Pain intensity was recorded every minute during testing. sICAM-1 concentrations in serum were determined by ELISA before, immediately after, and 60 min after test termination. Among all experimental groups, sICAM-1 significantly decreased immediately after pain induction. After 60 min, sICAM-1 concentrations returned towards initial values. Interestingly, a linear correlation was found between the extent of sICAM-1 changes and the initial concentrations. Whereas high initial values led to a distinct decrease of sICAM-1, low concentrations tended to increase. There was no statistically significant correlation between levels or alterations of serum sICAM-1 and pain intensity reported by the test subjects. In contrast to our previous findings in chronic pain patients, the present results show that sICAM-1 values do not correlate with the intensity of acute experimental pain. However, we were able to detect short-term changes of sICAM-1 after induction of nociceptive thermal stimuli, suggesting that this marker is part of a demand-oriented homeostatically controlled system.

## 1. Introduction

Pain is an experience comprising sensorimotor, mental and affective components. Despite the complexity of pain, the rated pain intensity is always assessed in daily clinical routine in order to adjust treatment. This is particularly true for acute, i.e., postsurgical pain. However, the assessment of pain may pose considerable problems for health professionals under certain conditions such as dementia. Since biomarkers for the quantification of pain do not (yet) exist, the type and extent of analgesic therapy is still determined by feedback and information given by the patients. Frequently used survey methods are scales developed to verbally, visually, or numerically record the intensity of perceived pain [1,2]. Though, this type of survey is not always reliable. Other methods are required to quantify pain for patients who are unable to communicate due to their age, their underlying disease, or anesthesia. Surrogate markers such as elevated blood pressure, increased heart rate, or groaning are unspecific and often result in inadequate pain therapy [3,4].

In the search for potential biomarkers to quantify pain intensity, some cytokines (e.g., IL-7, IL-13), free fatty acids, or hormones (e.g., glucagon, cortisol) could be identified whose serum concentrations showed alterations in the application of experimental pain models [5,6]. However, the potential biomarker described so far is very unspecific and often pathologically altered in the context of existing underlying diseases. In recent years, pain research has increasingly focused on pro- and anti-inflammatory molecules and their receptors. These play an important role in developing and maintaining inter alia neuropathic pain [7,8,9,10]. Our research group has investigated 30 of those cytokines and their serum levels for a possible association with the perceived intensity of pain in chronic pain patients (lasting from 6 months to several years and including neuropathic, nociceptive, and mixed pain). 

We found a significant correlation between both the initial values of sICAM-1 serum concentration and pain level as well as their respective changes [11]. 

An association between sICAM-1 and pain has been demonstrated in both animal models and patients with other types of pain (i.e., chest pain, migraine) [12,13].

In the present study, we therefore focused on the role and kinetics of sICAM-1 in acute experimental pain in healthy volunteers.

### 1.1. Intercellular Adhesion Molecule 1, ICAM-1

ICAM-1, also known as CD54 (cluster of differentiation 54), is a cell surface glycoprotein, which is typically expressed on endothelial cells and cells of the immune system (e.g., macrophages and lymphocytes) [14]. ICAM-1 is a ligand for MAC-1 (macrophage-1 antigen, also known as CR3) and LFA-1 (lymphocyte function-associated antigen, also known as CD11a/CD18), which in turn are located on the surface of leucocytes [15]. ICAM-1 mediates the adhesion of leukocytes to endothelial cells and is therefore an important component in leucocyte extravasation. In the course of inflammation or tissue injury, leukocytes migrate (cytokine-regulated) into the affected tissue and release vesicles with cytokines and endogenous opioids [16,17]. Thus, ICAM-1 plays not only an important role in inflammatory reactions but also in peripheral antinociception. It is known that the anti-inflammatory effects of opioids (endogenous or exogenous) are also caused by opioid-induced feedback mechanisms on leukocyte migration [18].

### 1.2. Soluble Intercellular Adhesion Molecule 1, sICAM-1

Soluble ICAM-1 can be generated either by proteolytic cleavage of the extracellular domain of membrane-bound ICAM-1 by human leucocyte elastase, cathepsin G, matrix metalloproteinase-1 and -9 and TACE/ADAM-17, or by alternative splicing [19,20,21,22]. Derived from an mRNA transcript, the expression of soluble ICAM-1 is modulated by cytokines such as IFNβ-1α [23]. In vitro studies point to a direct correlation between soluble ICAM-1 and membrane-bound ICAM-1, suggesting that increased levels of sICAM-1 are indicative of upregulation of membrane-bound ICAM-1 [24].

The present study was designed to investigate the role and kinetics of serum sICAM-1 in acute pain. Therefore, thermal nociceptive stimuli were induced using a device for quantitative sensory testing. It was examined whether the absolute concentrations and/or the extent of alteration of the initial concentrations correlated with the reported pain intensity.

## 2. Materials and Methods

### 2.1. Acute Pain Model

Since ICAM-1 plays a critical role in inflammatory processes of any kind, it was essential to conduct the study without the most significant possible bias. Participants with possible confounding factors such as psychiatric diagnosis, acute or chronic physical diseases and current medication were excluded in advance. 

**Figure 1:**
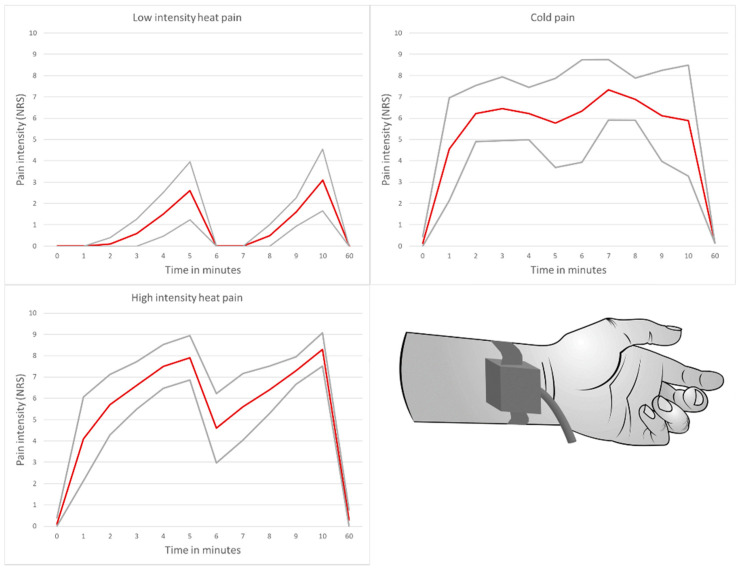
Average pain intensity induced by three different pain models developed by our research group. Thermal nociceptive stimuli were applied by a device for quantitative sensory testing [25]. The red line represents the average perceived pain intensity, the gray lines show the standard deviation.

To induce experimental pain in healthy subjects, we first established an appropriate acute pain model [25]. By using a device for quantitative sensory testing (QST; TSA- II NeuroSensory Analyzer, Medoc Ltd. Advanced Medical Systems Inc., Ramat Ishai, Israel), nociceptive heat and cold stimuli were set via the thermode at the volar forearm (see Figure 1). Because of the risk of thermal tissue damage depending on the temperature of the thermode and the duration of the stimulus interval, the high-intensity heat pain model was combined with topical capsaicin (commercial product, 750 µg/g), which causes a neurogenic inflammation with hyperalgesia and thus significantly lowers the pain threshold for heat stimuli [26]. Capsaicin treatment did not induce pain per se; the purpose of the application was solely to enhance nociceptive heat stimuli. 

The acute pain model included three experimental groups [25]:

1. Low-intensity heat pain model with application of a placebo cream
2. High-intensity heat pain model with application of a capsaicin cream
3. Cold pain model with application of a placebo cream

Moreover, the model was characterized by the following features:

1. Induction of a significant pain stimulus with an average perceived pain intensity of ≥6 on a numerical rating scale in the high-intensity heat pain and cold pain model over a test duration of 10 min
2. High tolerance of the pain model by the study participants
3. Avoidance of severe or irreversible tissue damage

The pain models were programmed to maintain a temperature range of 0–50 °C and switch off automatically after 5 min. The test subjects were able to interrupt the program at any time in case of pain intolerance utilizing a remote control. 

The perceived pain intensity was recorded right before the test, every minute during the experiment, and finally 60 min after the first survey via a numeric rating scale. 

#### 2.1.1. Test Preparation

The test areas on both volar forearms (about 2 cm proximal of the wrist crease) were prepared according to the randomised assignment. First, the skin was cleaned with an alcohol-based disinfectant. A hazelnut-sized amount of a commercial moisturiser (cold pain group and low-intensity heat pain group) or capsaicin cream (750 µg/g, high-intensity heat pain group) was then administered to an area of about 4 × 4 cm. An occlusive dressing was applied to enhance the effect. After 20 min, the dressings and cream residues were removed. The thermode was first attached to the right arm. After running through the assigned pain program, the thermode was changed to the contralateral arm to avoid thermal skin damage. 

In order to ensure the greatest possible exclusion of factors influencing pain perception, the experiments were always carried out between 4 and 7 p.m. at an ambient temperature of 21–23 °C (degrees Celsius). The experiments were always carried out by the same male investigator. 

#### 2.1.2. Low-Intensity Heat Pain Model

The low-intensity heat pain group underwent the same program as the high-intensity heat pain model but instead of a capsaicin cream, a commercial moisturizing cream (placebo cream) was applied to the test area. The previous topical application of capsaicin in the heat pain group resulted in the same heat stimulus being perceived as significantly more painful. This setup was designed to detect whether changes in sICAM-1 serum levels are due to the application of the chemical agent capsaicin and if the extent of the perceived pain intensity correlates with absolute concentrations or concentration changes of sICAM-1 [25].

#### 2.1.3. High-Intensity Heat Pain Model

The high-intensity heat pain model started at a thermode temperature of 32 °C (baseline). The temperature increased at a rate of 10 °C/s up to the first heat stimulus of 38 °C. The stimulus duration was 20 s each. After returning to the baseline temperature for a period of 0.5 s the next stimulus induction started. The temperature of the thermode increased stepwise by 1 °C after each heat impulse up to the maximum temperature of 50 °C. The program ended after five minutes. To prolong the pain induction without the risk of severe tissue damage, the program was restarted on the contralateral arm of the test subject [25]. 

#### 2.1.4. Cold Pain Model

The cold pain model also started with a thermode temperature of 32 °C. The temperature decreased at a rate of 10 °C/s to the first cold stimulus of 2 °C. The stimulus duration was 40 s. Subsequently, the thermode temperature returned to 15 °C (baseline) for 4 s before a new stimulus induction was started. The temperature of the thermode was lowered stepwise by 0.5 °C after each cold impulse to the minimum temperature of 0 °C. After five minutes, the program switched off and another test run was performed on the contralateral arm [25]. 

### 2.2. Subjects

For the experimental study, healthy, adult, pain-free test subjects were recruited. The experiment involved 15 women (age: mean = 29.3 years; SD = 7.4) and 15 men (age: mean = 29.1 years; SD = 5.0). Exclusion factors were pregnancy and any type of acute or chronic physical or psychological disease. One subject had to be excluded afterward due to pathological hypaesthesia.

### 2.3. Blinding

The experimental participants were randomly assigned to three experimental groups of 10 subjects each. There was no cross-over-design. To achieve double-blinding, the preparation of the subjects and anonymization of the pain programs were carried out by an assistant investigator. 

### 2.4. Assessment

Before the start of the trial, a standardized questionnaire was used to collect data from each subject regarding age, gender, education, weight, height, current and past illnesses, and medication intake over the last four weeks. 

The individual stress level was assessed using the German version of the Questionnaire for Actual Demands (KAB: Kurzfragebogen zur aktuellen Beanspruchung). By means of a 6-point scale ranging from 1 to 6 based on normalized adjectives (tensed–calm; solved–apprehensive; concerned–carefree; restless–relaxed; skeptic–trustful; uncomfortable–cozy), the KAB score was developed for assessing acute or chronic distress. Higher values indicate increased levels of distress [27]. Cut off value for exclusion was KAB ≥ 4. A numerical pain rating scale (NRS) ranging from 0 to 10 was used to measure the perceived pain intensity, in which 0 indicates no pain and 10 greatest conceivable pain.

### 2.5. Cytokine Measurement

Before, at the end and one hour after starting the experiment a sterile, a venous blood sample was taken from each test subject from the median cubital vein (EDTA sample tubes). Since it is unclear whether an inserted venous catheter might influence the serum concentration of sICAM-1, blood samples were taken by single punctures from alternating sides. Within 30 min the blood samples were centrifuged at 1000× *g* for 15 min. The blood plasma was then pipetted into plastic aliquots tubes (specimen á 150 µL) and immediately frozen at −80 °C. For the quantitative determination of sICAM-1 concentrations a Human ICAM-1/CD54 Allele-specific immunoassay (Quantikine^®^ ELISA, R&D Systems Europe, Ltd., Abingdon OX14 3NB, UK) was used. Quality check and double measurements were performed. 

### 2.6. Statistical Methods 

SPSS Version 25 of IBM Statistics was used for statistical analysis and randomization of the experimental groups. The data were tested for normal distribution using the Kolmogorov–Smirnov test. Differences in plasma sICAM-1 concentrations were tested for significance using the t-test for paired samples. Correlation coefficients were determined according to Pearson. Whether the mean values of the collected data differ depending on the experimental group was determined by univariate analysis of variance with repeated measurements and post hoc test (LSD). An a priori sample size calculation was performed with G-Power. We performed a power analysis based on our data of chronic pain patients with an observed effect size of 6.68. Since the standard deviation of the differences in chronic pain patients varied depending on the perceived pain level, we chose the largest observed standard deviation as the basis for calculation for the final case number estimate (effect size 3.18; sample size 9.13 for each experimental group). 

## 3. Results

### 3.1. Pain Intensity and Duration

The experimental design described above allowed the induction of a significant heat and cold pain stimulus with an average perceived pain intensity of NRS ≥ 6. In the low-intensity heat pain group, the average pain intensity over a 10-min test duration was NRS = 1 (see Figure 1). There was a significant difference in perceived pain intensities between the high-intensity pain groups (high-intensity heat pain and cold pain group) and the low-intensity heat pain group (*p* < 0.001), but not within the high-intensity pain groups (*p* = 0.64). 

### 3.2. Serum Levels of Soluble ICAM-1

**Figure 2:**
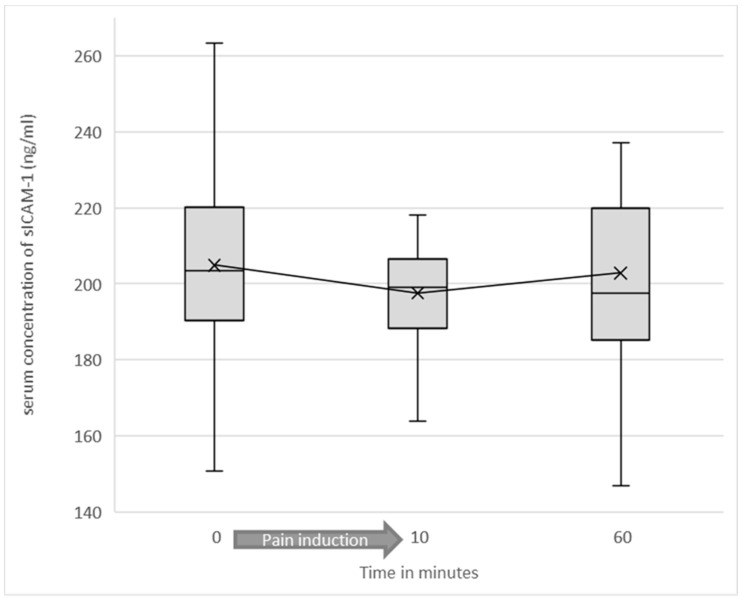
Soluble ICAM-1 serum concentrations as a function of test duration among all test participants are shown as a box plot diagram. Outliers are not shown. The difference in mean sICAM-1 concentration before and immediately after induction of pain was statistically significant (*p* = 0.003).

**Figure 3:**
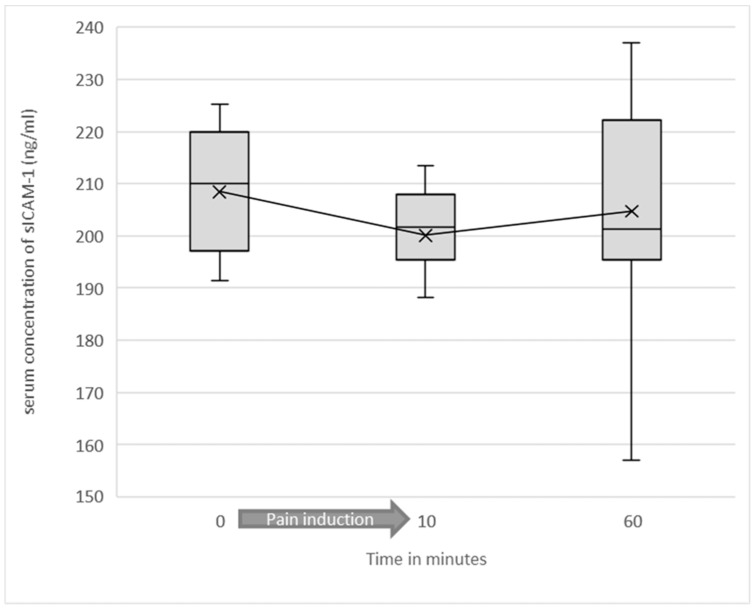
Soluble ICAM-1 serum concentrations as a function of test duration in the low-intensity heat pain group are shown as a box plot diagram. Outliers are not shown. The difference in sICAM-1 concentration before and immediately after heat induction was statistically significant (*p* = 0.037).

Preliminarily, it was tested whether the induction of pain (regardless of entity and intensity) caused a significant alteration of sICAM-1 concentrations across all test participants. Mean sICAM-1 concentration before the start of the experiment was 204.89 ng/mL (SD = 35.46). Immediately after induction of pain, mean sICAM-1 was 197.5 (SD = 27.13). At 60 min after starting the experiment, mean sICAM-1 value was 202.79 ng/mL (SD = 29.76). The difference in mean sICAM-1 concentration before and immediately after induction of pain was statistically significant (*p* = 0.003) (see Figure 2 and Figure 3). 

To test whether the alteration in sICAM-1 concentration depends on the type of pain (heat or cold) or pain intensity (high-intensity or low-intensity), three different statistical analyses were performed.

In the low-intensity heat pain group, mean sICAM-1 value before the start of the experiment was 208.43 ng/mL (SD = 28.76). Immediately after the end of heat induction, mean sICAM-1 value was 200.08 ng/mL (SD = 22.11). The difference in sICAM-1 concentration before and immediately after heat induction was statistically significant (*p* = 0.037). At 60 min after starting the experiment mean value of sICAM-1 concentrations nearly approached the baseline with 204.67 ng/mL (SD = 23.43). In the low-intensity heat pain group, no statistically significant correlation between the pain intensities perceived and the serum concentrations of sICAM-1 or changes in these concentrations could be found. (see Figure 3) 

In the high-intensity (capsaicin-enhanced) heat pain group, mean sICAM-1 value before starting the experiment was 194.33 ng/mL (SD = 23.79). Immediately after heat induction, mean sICAM-1 value was 189.47 ng/mL (SD = 18.61). The difference in sICAM-1 concentration before and immediately after the pain induction was not statistically significant (*p* = 0.176). After the trial, mean sICAM-1 concentration in serum approached the baseline value. The mean value of the sICAM-1 concentrations 60 min after starting the experiment was 193.04 ng/mL (SD = 19.51).

**Figure 4:**
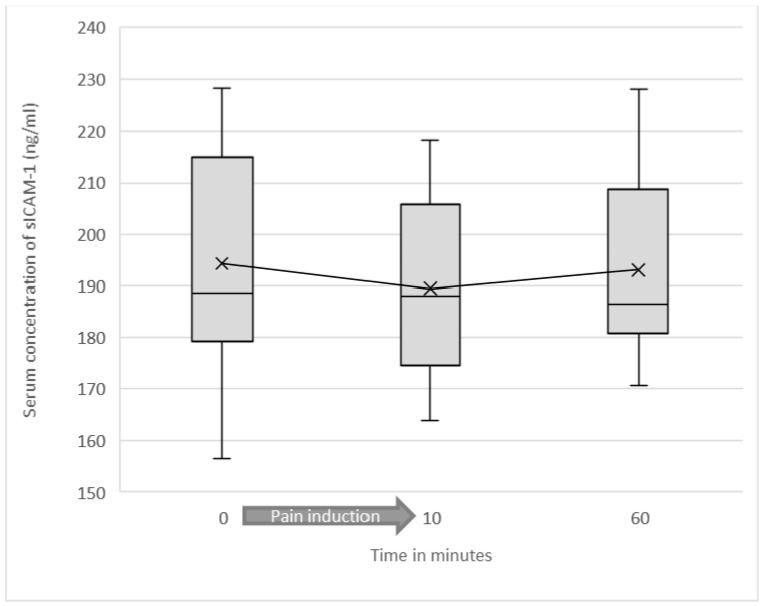
Soluble ICAM-1 serum concentrations as a function of test duration in the high-intensity heat pain group are shown as a box plot diagram.

In the high-intensity pain group, no statistically significant correlation between the pain intensities perceived and the serum concentrations of sICAM-1 or changes in these concentrations could be found (see Figure 4).

In the cold pain group, mean sICAM-1 value before starting the experiment was 212.69 ng/mL (SD = 51.08). Immediately after the end of pain induction, mean sICAM-1 value was 203.68 ng/mL (SD = 38.81). The difference in sICAM-1 concentration before and immediately after pain induction was not statistically significant (*p* = 0.120). After the trial, mean sICAM-1 concentration in serum approached the baseline value. The mean sICAM-1 concentration 60 min after starting the experiment was 211.53 ng/mL (SD = 42.94). 

**Figure 5:**
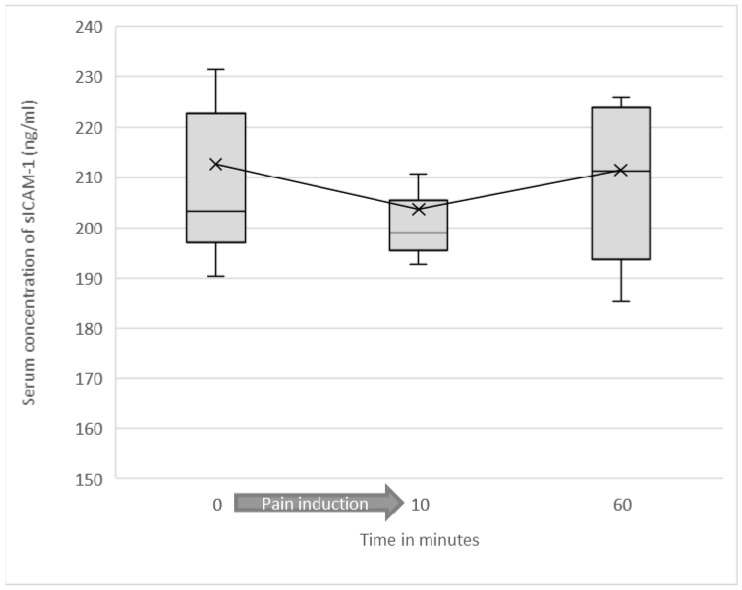
Soluble ICAM-1 serum concentrations as a function of test duration in cold pain group are shown as a box plot diagram. Outliers are not shown.

In the cold pain group, no statistically significant correlation between the pain intensities perceived and the serum concentrations of sICAM-1 or changes in these concentrations could be found (see Figure 5).

**Figure 6:**
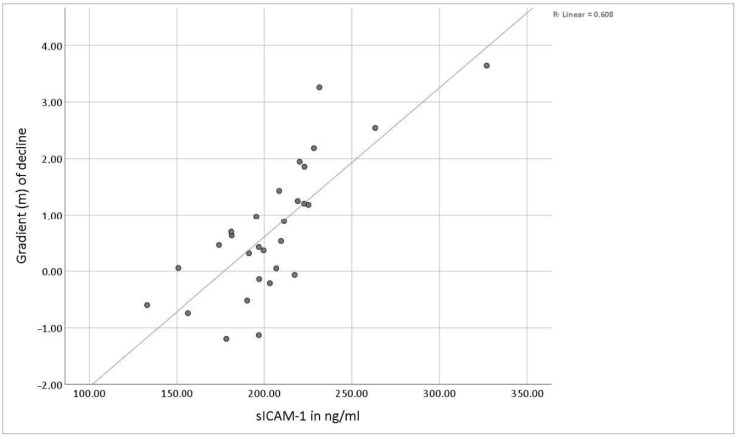
Illustration of the gradient of the decline in the sICAM-1 concentration after passing through the experimental setup in relation to the initial sICAM-1 concentration. The values of all test participants are shown.

Interestingly, we found that the extent of the decrease or increase of mean sICAM-1 values correlated with the level of the initial sICAM-1 concentration (correlation coefficient according to Pearson: 0.78; *p* < 0.01). Low initial concentrations even led to an increase of sICAM-1 in serum after running through the experiment (see Figure 6).

### 3.3. Sex-Related Differences

No significant sex-related differences in perceived pain intensities, baseline concentrations, or changes in concentrations of sICAM-1 could be found. A study by Riley et al. determined that at least 41 subjects must be examined to generate a sufficient statistical power to detect these differences [28].

## 4. Discussion

Alterations of sICAM-1 serum concentrations have already been related to various underlying diseases. They can be associated with viral infections, autoimmune diseases, cardiovascular diseases, and neurological or psychiatric disorders such as migraine or schizophrenia [29,30].

Consequently, the present study was designed to investigate the role and kinetics of sICAM-1 in acute pain. Experimental pain was induced in healthy test subjects using a device for quantitative sensory testing. Thermal nociceptive stimuli are perceived via different receptors. Heat stimuli primarily activate TRPV1 and TRPV2 receptors, whereas cold nociceptive stimuli predominantly activate TRPA1 and TRPM8 receptors [31,32]. Thus, in the present study, both types of pain were used to generate a significant pain level of NRS ≥ 6 for 10 min. 

According to the present findings, one might expect that the induction of acute pain or neurogenic inflammation caused by the application of capsaicin would lead to an increase of sICAM-1. However, we predominantly observed an initial drop in the sICAM-1 concentrations followed by an approximation to the base levels of sICAM-1 at 60 min after the start of the experiment. Across all experimental groups, the initial decrease was highly significant. A detailed analysis of the subgroups revealed that only in the low-intensity heat pain group the drop in the sICAM-1 was statistically significant, suggesting that neither the intensity nor the type (heat pain or cold pain) of pain is responsible for the obtained results. However, it cannot be excluded that this observation might also be related to the large standard deviation of the initial sICAM-1 values in the healthy subjects. 

In contrast to our previous findings concerning chronic pain patients, there was no correlation between sICAM-1 concentration and pain intensity in the present study, indicating that measurement of this marker is not suitable for the quantification of acute pain. The observed differences in the role of sICAM-1 in acute and chronic pain may be due to different pathophysiological mechanisms underlying the two entities. In particular, dysfunction of the hypothalamic-pituitary-adrenal (HPA) axis often described in chronic pain patients should be considered because of its extensive influence in the expression and function of immune cells and adhesion molecules. Immunocompetent cells (e.g., monocytes or lymphocytes) exhibit receptors for neuroendocrine products of the HPA axis (e.g., CRH, cortisol). The imbalance of the HPA axis and its neurotransmitters and resulting long-term alterations, such as downregulation or increased resistance of targeted receptors (e.g., glucocorticoid receptors), lead to changes in trafficking adhesion, proliferation and cytokine secretion of immune cells [33,34,35,36].

In the experimental setup described here, high initial values of sICAM-1 distinctly decreased, whereas low concentrations tended to increase due to pain induction. In both cases, an approximation towards initial values was seen at the 60-min time point. The increase or decrease of sICAM-1 serum concentrations and the extent of alteration depended on the baseline concentrations. 

Since the soluble form of ICAM-1 is also a ligand of LFA-1-expressing leukocytes, the observed attenuation might be explained by receptor-binding, followed by an increase through proteolytic cleavage of upregulated membrane-bound ICAM-1, alternative splicing or dissolving from any receptor targeted by sICAM-1. The mechanism of response regulation takes place with a latency. However, according to this theory, an initial decrease in sICAM-1 would be expected among all experimental subjects. Considering the linear relationship between baseline and extent of alteration of sICAM-1 concentration, the changes seem to be demand-oriented as a possible expression of immunological homeostasis. This mechanism could allow an adequate immune reaction to be triggered or an overshooting response to be prevented. The results obtained in this study suggest a balanced and rapidly adaptable system of circulating immunomodulatory proteins, including sICAM-1. They also provide information about the short-term kinetics of sICAM-1 after experimental pain induction for the first time. The regulating mechanism responsible for the changes in sICAM-1 concentrations described here needs to be investigated in future studies.

The fact that the abovementioned results could be obtained in all experimental groups reveals an important limitation of the present study design: it cannot be conclusively answered whether the thermal stimulus, the skin penetration caused by blood sampling, or even simply the induction of acute stress led to the changes of sICAM-1 concentrations. 

It has to be taken into account that pain is a complex experience, including physical, psychological, and social aspects. This is particularly true for chronic pain, which the “bio-psycho-social pain model” commonly defines. However, psychological stress is also an essential part of acute nociception. Therefore, it can never be ruled out that changes observed in acute and chronic pain are stress-induced. In fact, it has been shown that stress and mental distress influence the expression of adhesion molecules. Heinz et al. observed an increase of sICAM-1 concentration two hours after the induction of acute psychological stress followed by normalization on the following day [37]. Therefore, even the expectation of a potentially painful event could have induced the changes of sICAM-1 in our study. 

In further studies providing a more extended observation period, it will be of interest to evaluate whether the nociceptive stimuli, in contrast to, e.g., acute mental stress, cause a long-term upregulation of membrane-bound and soluble ICAM-1 as a possible sign of increased peripheral antinociception. 

In conclusion, we found that in contrast to our previous results in chronic pain, sICAM-1 levels do not correlate with the intensity of acute experimental pain. However, we were able to demonstrate the short-term kinetics of this adhesion molecule after induction of painful stimuli in healthy volunteers for the first time. Our data suggest that sICAM-1 is part of a demand-oriented homeostatically controlled system involved in the experience of pain. 

## Data Availability

The data presented in this study are available on request from the corresponding author. The data are not publicly available as they are part of the ongoing doctoral process of the author P.Lüke.
